# Supplementary figures and images for: Vitronectin-derived bioactive peptide prevents spondyloarthritis by modulating Th17/Treg imbalance in mice with curdlan-induced spondyloarthritis
Source: PLoS One. 2022 Jan 5;17(1):e0262183. doi: 10.1371/journal.pone.0262183 (PMC8730421; doi:10.1371/journal.pone.0262183)

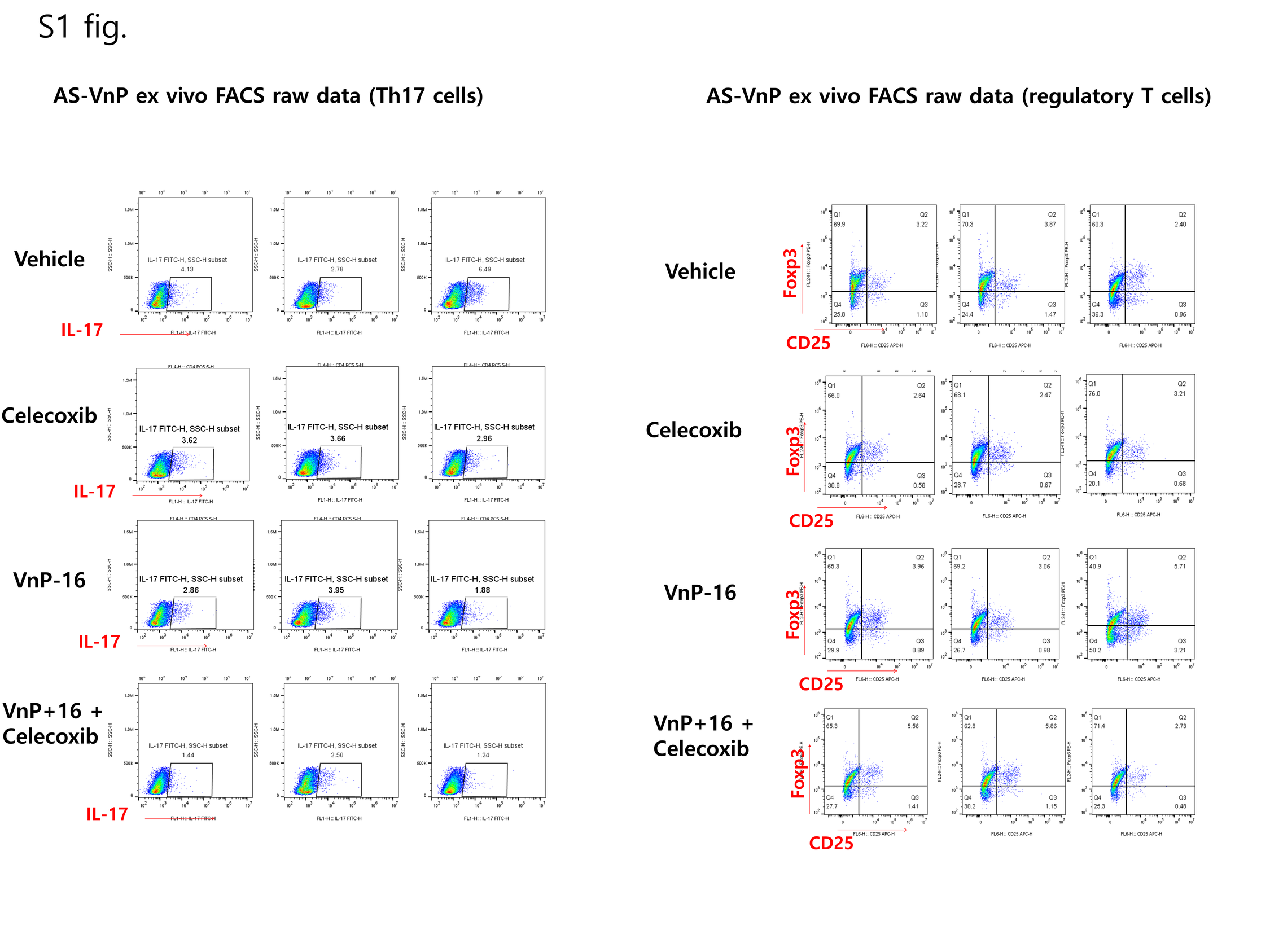

Supplement: S1 Fig — (TIF) [file pone.0262183.s001.tif]

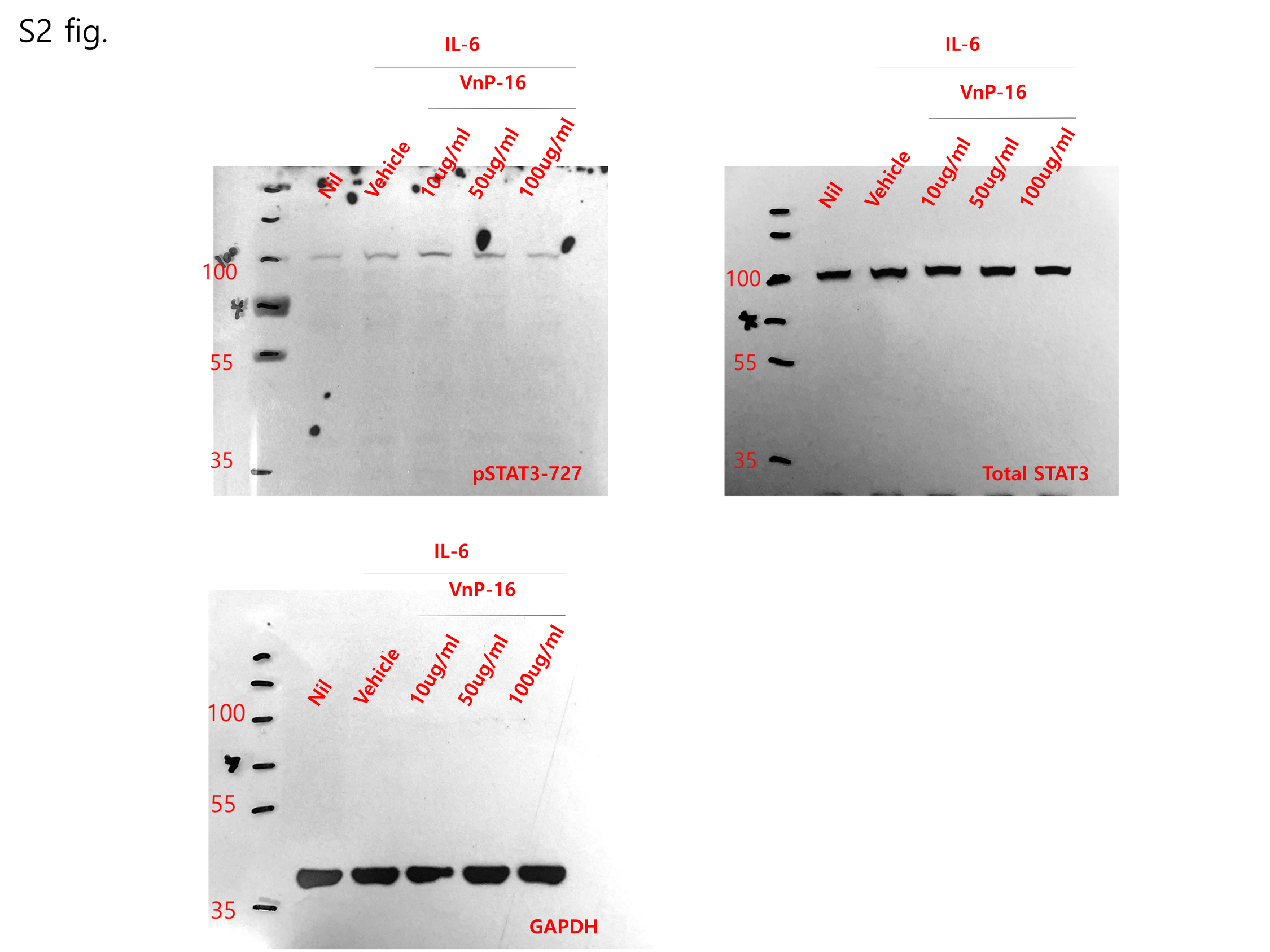

Supplement: S2 Fig — (TIF) [file pone.0262183.s002.tif]
